# Current approaches to immunotherapy in noncolorectal gastrointestinal malignancies

**DOI:** 10.6061/clinics/2018/e510s

**Published:** 2018-10-06

**Authors:** Victor Hugo Fonseca de Jesus, Tiago Cordeiro Felismino, Milton José de Barros e Silva, Virgílio de Souza e Silva, Rachel P Riechelmann

**Affiliations:** I Departamento de Oncologia Médica, A.C. Camargo Cancer Center, Sao Paulo, SP, BR; II Departamento de Radiologia e Oncologia, Instituto do Cancer do Estado de Sao Paulo (ICESP), Hospital das Clinicas HCFMUSP, Faculdade de Medicina, Universidade de Sao Paulo, Sao Paulo, SP, BR

**Keywords:** Immunotherapy, Gastrointestinal, Neoplasms

## Abstract

Noncolorectal gastrointestinal (GI) malignancies are among the most frequently diagnosed cancers. Despite the undeniable progress in systemic treatments in recent decades, further improvements using cytotoxic chemotherapy seem unlikely. In this setting, recent discoveries regarding the mechanism underlying immune evasion have prompted the study of molecules capable of inducing strong antitumor responses. Thus, according to early data, immunotherapy is a very promising tool for the treatment of patients with GI malignancies. Noncolorectal GI cancers are a major public health problem worldwide. Traditional treatment options, such as chemotherapy, surgery, radiation therapy, monoclonal antibodies and antiangiogenic agents, have been the backbone of treatment for various stages of GI cancers, but overall mortality remains a major problem. Thus, there is a substantial unmet need for new drugs and therapies to further improve the outcomes of treatment for noncolorectal GI malignancies. “Next-generation” immunotherapy is emerging as an effective and promising treatment option in several types of cancers. Therefore, encouraged by this recent success, many clinical trials evaluating the efficacy of immune checkpoint inhibitors and other strategies in treating noncolorectal GI malignancies are ongoing. This review will summarize the current clinical progress of modern immunotherapy in the field of noncolorectal GI tumors.

## INTRODUCTION

Gastrointestinal (GI) cancers represent the most common malignancy worldwide 1. Colorectal, gastric and liver cancers rank second, third and fourth in cancer-related mortality, respectively 2. Although early-stage GI cancers are amenable to surgical resection with curative intent, 25-50% of patients develop metastatic disease during follow-up. Moreover, approximately 25% of GI cancers are diagnosed at an advanced, incurable stage 3,4.

Despite the recent progress in diagnosis and treatment, including the introduction of targeted therapies, patients with advanced GI cancer still fare particularly poorly 5,6. In the last years, advances in the understanding of the intersection between immune surveillance and tumor growth have led to broad therapeutic advances in many cancer types. For example, we observed significant improvements in the overall survival and treatment response duration in some solid tumors, such as melanoma, non-small cell lung cancer and genitourinary cancers, as well as in hematologic malignancies 7. Additionally, the recognition of some aspects of the pathogenesis of GI malignancies has rendered immunotherapy a promising tool in the management of GI cancer.

The idea that at least some GI cancers are “immunogenic enough” to be treated in clinical trials of immune-directed therapies initially arose from the observation that chronic infections and the associated inflammation are well-known risk factors for some GI cancers. For example, hepatitis B and C (HCV) are related to liver cancer, and *Helicobacter pylori* is a major risk factor for gastric adenocarcinoma. Irritants such as tobacco or asbestos are not only carcinogenic but can also trigger chronic inflammation. Finally, inflammatory bowel disease significantly increases colorectal cancer risk. Inflammation generally leads to genomic instability, accompanied by the accumulation of mutations and neoantigens. Reinforcing these findings, vast epidemiological data suggest that aspirin, which is a well-known anti-inflammatory agent, has protective antitumor effects against several cancer types, including colorectal cancer 8.

Considering these aspects of GI cancer pathogenesis and the impressive results observed in other solid tumors, several studies have been conducted to assess the activity of next-generation immunotherapy in patients with digestive tract tumors. Despite the relatively slow pace of immunotherapy development in GI cancers, initial results suggest that this approach may be effective 9. Preliminary results from phase 1 and 2 trials in esophageal, gastric and hepatobiliary cancer report response rates ranging from 15% to 25%, similar to the response rates described for other malignancies 10. Given the recent abundance of information on immunotherapy in GI tumors, we conducted a comprehensive review of the clinical trials evaluating immune-directed therapies in noncolorectal GI cancers, with a focus on immune checkpoint (ICP) inhibitors.

## CLINICAL EVIDENCE OF IMMUNOTHERAPY IN NONCOLORECTAL GASTROINTESTINAL MALIGNANCIES

### Esophageal Squamous Cell Carcinoma

In patients with advanced esophageal squamous cell carcinoma (SCC), immunotherapy has shown promising results. Table 1 summarizes the data from prospective trials of ICP inhibitors conducted in esophageal cancer. KEYNOTE-028 is a phase 1b study that tested the anti-programmed cell death protein 1 (PD-1) monoclonal antibody pembrolizumab (10 mg/kg every two weeks) in 83 patients with advanced esophageal squamous carcinoma and adenocarcinoma that presented positive immunohistochemical expression of programmed cell-death receptor ligand 1 (PD-L1). In a heavily pretreated population (87% of whom had undergone ≥2 prior therapies for metastatic disease), the overall response rate was 30.4% (29.4% in SCC). Some tumor shrinkage was observed in 52.2% of the patients, and the side effects were manageable. Only four patients experienced grade 3 treatment-related adverse events; these events included lymphopenia, anorexia, liver disorder and generalized rash 11.

An early uncontrolled trial (ONO-4538-07) of nivolumab (3 mg/kg every two weeks), which is another anti-PD-1 agent, in refractory metastatic esophageal SCC (irrespective of positive immunochemical PD-L1 expression) showed objective responses in 11 of 64 assessable patients (17%). The median time to progression was 2.8 months, and the median overall survival time was 10.8 months. Again, the safety profile was expected and manageable 12.

### Esophagogastric Adenocarcinoma

Early attempts to employ immunotherapy in gastric cancer were disappointing. Interferon (IFN) activity could not be demonstrated in either the adjuvant or metastatic settings 13,14. Similarly, early trials using vaccines in the setting of advanced disease failed to demonstrate any benefit. Nevertheless, later vaccine trials indicated that patients who could mount a tumor-specific immune response had better survival than those who could not 15,16. Thus, there is a subgroup of patients who might benefit from immunotherapy, and this characteristic is being explored in recent trials of ICP inhibitors.

#### Checkpoint Inhibitors

Table 2 summarizes the current data on ICP inhibitors in gastric cancer. The activity of pembrolizumab in advanced gastric cancer was first tested in the phase 1b trial KEYNOTE-012. In this study, among 39 patients treated with pembrolizumab (10 mg/kg every two weeks), the objective response rate was 22%, the median progression-free survival time was 1.9 months and the median overall survival time was 11.4 months 17.

KEYNOTE-059 is a multicohort trial testing pembrolizumab in patients with advanced gastroesophageal adenocarcinoma 18. Cohort 1 consisted of 259 patients who had undergone at least two prior lines of therapy. All patients received 200 mg intravenous (IV) pembrolizumab every three weeks. In cohort 1, 57% of the patients were PD-L1 positive. The response rate was 12% in the overall population (16% in PD-L1-positive patients and 6% in PD-L1-negative patients); complete responses were observed in 3% of the patients, regardless of tumor PD-L1 status. The median overall survival time was 5.5 months. Grade 3-5 adverse events occurred in 18% of the patients and included two deaths. Five patients experienced grade 3 immune-mediated adverse events. Microsatellite instability (MSI) was investigated in 174 patients, of whom 4% presented high-frequency MSI (MSI-High); the objective response rate was 57.1% in the MSI-High patients but only 9.0% in the non-MSI-High group 19. Based on these data, the Food and Drug Administration (FDA) approved pembrolizumab for use in patients with PD-L1 positive metastatic esophagogastric adenocarcinomas after two other lines of treatment had been attempted.

KEYNOTE-059 cohort 2 consisted of 25 untreated PD-L1-unselected patients treated with chemotherapy (cisplatin and fluoropyrimidine) plus pembrolizumab. Of the tumors, 64% were PD-L1-positive. The response rate was 60% in the entire cohort (69% in PD-L1-positive patients and 38% in PD-L1-negative patients), and complete responses were observed in 4% of the patients (all with PD-L1-negative tumors). The median overall survival time was 13.8 months. Cytopenias attributed to chemotherapy were the main toxicities reported. Grade 3 immune-mediated adverse events were observed in 16% of patients (mainly skin toxicity) 18. Cohort 3 recruited 31 newly diagnosed PD-L1-positive, HER-2-negative patients. All received pembrolizumab monotherapy as a first-line regimen, with promising results: an overall response rate of 26% and a median overall survival time of 20.7 months. Adverse events occurred in 77% of the patients (a 23% incidence of grade 3-5 adverse events) 18.

CheckMate 032 20 is a phase 1/2 trial that enrolled 160 Western patients pretreated (> 1 line) for advanced gastroesophageal cancer. The patients were distributed into three different groups. The primary endpoint was the overall response rate, and no statistical comparisons were made across the groups. In cohort 1, 59 patients received 3 mg/kg nivolumab every two weeks, and 38% of the tumors were PD-L1-positive. The overall response rate and complete response rate were 12% and 2%, respectively, and the median overall survival time was 6.2 months. Grade 3-4 serious adverse events occurred in 5% of the patients. In cohort 2, 49 patients received 1 mg/kg nivolumab plus ipilimumab, which is an anti-cytotoxic T lymphocyte-associated protein 4 (CTLA4) monoclonal antibody, at a dosage of 3 mg/kg every three weeks; 24% of the tumor samples were PD-L1 positive. The overall response rate was 24%, and the median overall survival time was 6.9 months; 35% of the patients experienced grade 3-4 serious adverse events. Finally, in cohort 3, in which 30% of the tumor samples were PD-L1-positive, 52 patients received 3 mg/kg nivolumab plus 1 mg/kg ipilimumab every three weeks. The response rate was 8%, and the median overall survival time was 4.8 months. Grade 3-4 serious adverse events occurred in 17% of the patients.

The recently published phase 3 trial ATTRACTION-2 included 493 Asian patients with advanced gastric and esophagogastric junction adenocarcinoma who had been treated with at least two prior lines of treatment; these patients were randomized for treatment with 3 mg/kg nivolumab every two weeks or with placebo 21. No patient selection based on PD-L1 expression or mismatch repair proficiency status was conducted. The median overall survival time was 5.26 months in the nivolumab arm and 4.14 months in the placebo arm (HR = 0.63, *p* < 0.0001). The twelve-month overall survival rates in the nivolumab and placebo arms were 26.2% and 10.9%, respectively. In this study, PD-L1 expression did not predict survival benefit. Toxicity of any grade occurred in 43% of the patients in the nivolumab arm (grade 3-4 toxicity occurred in 10%). This trial is the first phase 3 trial showing the survival benefit of immunotherapy in esophagogastric cancer, and based on these results, nivolumab was approved in Japan in September 2017 for use in patients with esophagogastric adenocarcinoma who have undergone two or more prior lines of treatment.

In 2017, the Food and Drug Administration (FDA) approved pembrolizumab for patients with mismatch repair-deficient (dMMR) tumors based on the impressive results achieved in a prospective phase 2 study; this approval is the FDA's first tissue/site-agnostic approval 22. In this trial, 86 previously treated patients with Lynch syndrome-related dMMR tumors (mostly colorectal, but the trial included five patients with esophagogastric cancer) received pembrolizumab monotherapy. The objective response rate was 40% in the colorectal cohort and 57% (4 of 7 patients) in the noncolorectal cohort. The average time to response was 21 weeks, and the median overall survival time had not yet been reached. Toxicity was considered mild, but endocrine disorders were observed in 21% of patients.

The phase 1b study JAVELIN assessed the activity of the anti-PD-L1 monoclonal antibody avelumab (10 mg/kg every two weeks) in 151 patients with advanced esophagogastric cancer (as either second-line therapy or maintenance therapy after first-line therapy) 23. The unconfirmed response rate was 9.7% and 9.0% in the second-line and maintenance groups, respectively. Among PD-L1-positive patients in the second-line setting, the response rate was 18.2%. Grade 3 adverse events occurred in 9.9% of the patients. Despite exciting early phase data, two recently published phase 3 trials did not show a clear survival gain with immunotherapy. In JAVELIN GASTRIC 300, 24 371 patients were randomized to avelumab (10 mg/kg every two weeks) or the physician's choice of chemotherapy (paclitaxel or irinotecan) or best supportive care after having undergone prior treatment with 2 or more lines of therapy; the patients achieved a median overall survival of 4.6 m *versus* 5.0 months (HR:1.1, CI 0.9 -1.4, *p*=0.81). The KEYNOTE-061 trial 25 randomized 592 patients who experienced progression with first-line platinum/fluoropyrimidine treatment to either pembrolizumab or standard-dose paclitaxel. Overall survival results were disappointing, with median overall survival times of 9.1 months for pembrolizumab and 8.3 months for paclitaxel (HR: 0.82, CI: 0.66 - 1.03, one-sided *p*=0.04). Post hoc analysis showed that patients whose tumors had CPS >10% or MSI-H experienced improved survival with pembrolizumab.

Importantly, recent data show that the activity of different classes of ICP inhibitors in esophagogastric cancer may vary. For example, anti-CTLA4 antibodies seem to have little, if any, activity in these tumors. An initial trial with the anti-CTLA4 monoclonal antibody tremelimumab showed disappointing results 26. Similarly, no survival benefit from maintenance ipilimumab therapy was seen in a recently published phase 2 trial 27 in which 114 patients with advanced gastroesophageal cancer who had achieved an outcome of at least stable disease after first-line treatment were randomized to ipilimumab treatment (10 mg/kg every three weeks for four doses, followed by 10 mg/kg every 12 weeks for up to three years) or best supportive care. The primary endpoint of immune-related progression-free survival (irPFS) in the ipilimumab arm was 2.92 months, *versus* 4.90 months in the comparator arm. These results led to study cessation.

## HEPATOPANCREATOBILIARY CANCER

### Pancreatic Cancer

#### Vaccines

Vaccines are the most frequently investigated form of immunotherapy in pancreatic cancer. Unfortunately, the results so far have been rather disappointing. The activity of the cell-based vaccine algenpantucel-L, which is derived from two irradiated pancreatic cancer cell lines (HAPa-1 and HAPa-2) transmuted to express the murine alpha-1,3-galactosyl-transferase enzyme, has recently been evaluated in a phase 3 trial (IMPRESS). In this trial, 722 patients were randomized to receive either algenpantucel-L in combination with standard of care or standard of care alone after pancreatic cancer resection. The median overall survival was 27.3 months in the algenpantucel-L arm and 30.4 months in the control arm (not significantly different) 28. In addition, two protein-based vaccines have been evaluated in the advanced disease setting. In the TeloVac trial, 1062 patients with locally advanced or metastatic pancreatic cancer were randomized to receive chemotherapy (capecitabine and gemcitabine), sequential chemoimmunotherapy or concurrent chemoimmunotherapy 29. A human telomerase reverse transcriptase catalytic subunit peptide vaccine was used as immunotherapy. No differences in overall survival time were observed (chemotherapy alone: 7.9 months; sequential chemoimmunotherapy: 6.9 months; concurrent chemoimmunotherapy: 8.4 months). Another large randomized trial evaluated the activity of the gastrin immunogen peptide-based vaccine G17DT in patients with pancreatic cancer unsuited for or unwilling to receive chemotherapy 30. In this trial, seventy-nine patients received treatment with G17DT, and 75 patients received placebo. Overall, no differences in survival were observed. In a post hoc analysis, patients who developed an anti-G17DT response experienced longer overall survival times than those who did not develop this response.

Recent trials have tried to boost vaccine effectiveness by using attenuated or killed bacteria as immune modulators. In an initial trial, 90 patients with metastatic pancreatic cancer were randomized (2:1) to immunotherapy using GVAX/cyclophosphamide in combination with CRS-207 (a live-attenuated formulation of mesothelin-expressing *Listeria monocytogenes*) or GVAX/cyclophosphamide alone 31. Patients treated with the immunomodulator had longer survival times than those who were not (6.1 *vs*. 3.9 months, respectively). Moreover, patients with enhanced mesothelin-specific CD8 T-cell responses experienced improved outcomes. Nevertheless, the ECLIPSE trial showed that this boosted immunotherapy regimen was no more effective than standard chemotherapy 32. In this study, 303 patients were randomized (1:1:1) to receive GVAX/cyclophosphamide + CRS-207, CRS-207 alone or chemotherapy (physician's choice). No differences in median overall survival times were observed (GVAX/cyclophosphamide + CRS-207: 3.8 months; CRS-207: 5.4 months; chemotherapy: 4.6 months). In another trial (IMAGE-1), 110 patients with locally advanced or metastatic pancreatic cancer were randomized (2:1) to receive gemcitabine + IMM-101 or gemcitabine alone 33. IMM-101 is an immunomodulator derived from heat-killed *Mycobacterium obuense*. Despite an improvement in the progression-free survival time in patients receiving the immunomodulator (HR = 0.58, 95% CI 0.37−0,91; *p* = 0.16), no differences were observed in the overall survival time (gemcitabine + IMM-101: 6.7 months; gemcitabine: 5.6 months). Thus, previous vaccine studies in pancreatic cancer did not achieve clinically meaningful results. However, the role of vaccine-mediated immunotherapy in pancreatic cancer cannot be ruled out, as some studies have shown that patients mounting an adequate response to immunotherapy survived longer than those who did not. As a result, a new generation of cancer-directed vaccine trials using recent immunological knowledge is underway.

#### Checkpoint Inhibitors

To date, ICP inhibitors have shown disappointing activity in pancreatic cancer. The efficacy of ipilimumab has been assessed in three prospective studies. In the first study, 27 patients with locally advanced or metastatic pancreatic cancer were treated with 3 mg/kg ipilimumab every three weeks for four cycles 34. One patient experienced a delayed partial response after initial disease progression. Additionally, minor responses were seen in two patients. The median overall survival time was approximately 20 weeks. A phase 1b trial evaluated the activity and safety of 10 mg/kg ipilimumab every three weeks for four cycles either alone or in combination with the cell-based vaccine GVAX 35. The median overall survival time was 3.6 months for ipilimumab monotherapy and 5.7 months for combination treatment (*p* = 0.072). No response according to RECIST was observed. Recently, ipilimumab was combined with gemcitabine in a small phase 1b trial 36. Among 16 patients, two experienced a partial tumor response and five experienced stable disease as best response. The median progression-free survival time was 2.5 months, and the median overall survival time was 8.5 months.

In addition, trials were performed to evaluate the role of anti-PD-1 or anti-PD-L1 antibodies in patients with pancreatic cancer. In a phase 1 study, 14 patients with pancreatic cancer were treated with the anti-PD-L1 antibody BMS-936559, but no responses were observed 37. An additional trial evaluated the activity of the anti-PD-L1 antibody durvalumab (MEDI-4736) in 25 patients with pancreatic cancer 38. Partial responses were observed in 3 patients, and the disease control rate (DCR) at 12 weeks was 21%. Finally, in a recent phase 1 study, 17 patients with pancreatic cancer were treated with nivolumab and nab-paclitaxel (arm A; n = 11) or with nivolumab, gemcitabine and nab-paclitaxel (arm B; n = 6). Responses according to RECIST were seen in both arms (arm A: two responses, arm B: three responses) 39.

The particularly disappointing results of the ICP inhibitor studies conducted so far in pancreatic cancer are the consequence of a complex interplay among the tumor cells, tumor microenvironment, microbiome and immune system 40. Pancreatic cancer cells can induce the expression of proteins (e.g., CTLA4, or PD-L1) and the secretion of cytokines (e.g., GM-CSF) that prevent effector T cells from infiltrating and destroying the tumor 40,41. Additionally, the tumor stroma acts like a barrier, creating a hypovascular and hypoxic environment that blocks the penetration of small molecules 42. Finally, the presence of certain bacteria has been shown to facilitate the development of pancreatic cancer, possibly by inducing an inflammatory state 43. The next generation of trials in pancreatic cancer will certainly address this complexity by targeting multiple tumor- and microenvironment-related pathways and exploring newer immunological strategies.

### Hepatocellular Carcinoma

Hepatocellular carcinoma (HCC), the most common primary hepatic tumor, develops from chronic inflammation induced by carcinogens, such as alcohol, or by infections, such as viral hepatitis B and HCV. Thus, immunotherapy might be effective in treating HCC. However, the results of the initial immunotherapy studies in HCC have not been very promising. Randomized trials evaluating the role of IFN in patients with advanced HCC 44,45 and in the adjuvant setting 46,47 had disappointing results. Another approach to immunotherapy in HCC is adoptive cell therapy. In randomized trials in an adjuvant setting, treatment with autologous cytokine-induced killer (CIK) lymphocytes led to improvements in recurrence-free survival and/or overall survival times 48-51. Despite some methodological issues in these studies, this approach has been proved feasible, and new trials evaluating these approaches to immunotherapy in HCC are underway.

#### Checkpoint Inhibitors

In a small phase 1/2 trial, 17 patients with advanced HCC secondary to HCV infection were treated with the anti-CTLA4 antibody tremelimumab at a dosage of 15 mg/kg every 90 days 52. The objective response rate was 17.6%, and 45% of the patients experienced disease control for at least 6 months. The activity and safety of the anti-PD-L1 antibody durvalumab was assessed in a phase 1 trial that enrolled 40 patients (93% of whom had been previously treated with sorafenib) 53. The objective response rate was 10.3%, and 33.3% of the patients experienced disease control for at least 6 months. Patients with cirrhosis secondary to HCV seemed to derive the greatest benefit from durvalumab. The largest study to date to investigate the efficacy of ICP inhibitors in HCC was the expanded cohort of the phase 1/2 CheckMate 040 trial 54, in which 262 patients with HCC (most of whom had been previously treated with sorafenib) were treated with nivolumab at a dosage of 3 mg/kg every two weeks. The overall objective response rate was 19%, with a median response duration of 9.9 months. Three patients (1%) experienced radiological complete response. The DCR was 64%, and 37% of the patients experienced disease control for at least 6 months. The median progression-free survival time was 4.0 months, and the median overall survival time had not been reached at the end of the trial. The efficacy outcomes were similar between subgroups of patients with viral hepatitis and alcohol-induced liver disease. These results led to the accelerated FDA approval of nivolumab in September 2017 as a second-line treatment of HCC. Similar results were shown with pembrolizumab in the KEYNOTE-224 55. In this phase II study, 104 patients with advanced HCC were treated with pembrolizumab 200 mg every 3 weeks. The overall response and disease control rates were 17 and 61%, respectively. The median progression-free and overall survival times were 4.9 and 12.9 months, respectively. In an attempt to improve the effectiveness of immunotherapy for HCC, 40 patients were enrolled in a phase 1 trial evaluating the activity of the combination of durvalumab and tremelimumab. The overall response rate was 20%, and the DCR at 16 weeks was 57.5% 56. Given the promising results of ICP inhibitors in HCC, further data are expected regarding combination immunotherapy for HCC treatment.

### Biliary Tract Cancer

Only one study has evaluated the role of ICP inhibitors in advanced biliary tract cancer. In KEYNOTE-028, 24 patients with PD-L1-positive tumors (≥ 1%) were treated with pembrolizumab at a dosage of 10 mg/kg every two weeks until confirmed progression or unacceptable toxicity occurred or until two years of treatment were completed. Partial response and stable disease were each seen in four patients. Among the responders, the treatment duration was longer than 40 weeks 57. Additionally, patients with biliary cancers were enrolled in the noncolorectal cohort in the phase 2 trial of pembrolizumab for the treatment of dMMR tumors, and the overall response rate of the cohort was 71% 58.

### Neuroendocrine Tumors

Neuroendocrine tumors (NETs) are a heterogeneous group of tumors that range from well-differentiated tumors, i.e., G1 and G2 NETs, to high-grade aggressive cancers (poorly differentiated G3 carcinomas). Immunotherapy has been restricted to well-differentiated tumors; IFN has the longest history of use. Studies of IFN-alpha monotherapy have generally shown tumor stabilization, measured by various methods, in 50 to 70% of patients with previously progressive GI NETs and have shown improvement in 30 to 70% of patients with carcinoid symptoms 59.

A recent phase 1b trial, KEYNOTE 028, tested pembrolizumab monotherapy in pretreated patients with well-differentiated carcinoid tumors (mostly midgut NETs) and pancreatic NETs 60. Approximately 20% of the 35 carcinoid tumors and 24.5% of the pancreatic NETs were PD-L1-positive. The overall response was 12% among the carcinoid tumors and 6% in the pancreatic NET cohort, but no complete responses were observed. The median progression-free survival time was less than 6 months in both groups, but durable responses were observed. The median response duration was 9.2 months for patients with carcinoid tumors and 20.3 months for patients with pancreatic NETs, and the median overall survival time was 20.1 months for both cohorts.

However, no study has evaluated immunotherapy for the treatment of high-grade NETs. This subgroup is heterogeneous and comprises NETs with a high ki67 index and poorly differentiated neuroendocrine carcinomas (NECs). Both subgroups are molecularly distinct; studies have shown that poorly differentiated NECs display molecular alterations that resemble their carcinoma/adenocarcinoma counterparts, such as mismatch repair deficiency, which has been observed in approximately 10% of gastric and colorectal NECs 61.

Therefore, although the further exploration of immunotherapy for the treatment of well-differentiated NET can be rationalized, the proper identification of immunogenic subgroups is crucial to prove the benefit of checkpoint inhibitors in NET treatment. For patients with NECs, a MSI-H or dMMR status should be determined; pembrolizumab is an effective treatment option for MSI-H- or dMMR-positive patients.

### Predictive Biomarkers

As previously shown, despite the relatively short progression-free survival time observed in most clinical trials, a subgroup of patients has experienced a durable tumor response in almost every study. However, few studies have evaluated predictive biomarkers for immunotherapy response in GI cancers. As a marker of PD-1/PD-L1 inhibitory pathway activation, PD-L1 has been analyzed by immunohistochemistry in many types of tumor specimens to try to predict the benefit of immunotherapy. Different antibodies have been used in studies evaluating anti-PD-1 or anti-PD-L1 antibodies, so the generalization of the resulting data may be flawed. Similarly, heterogeneity in tumor PD-L1 expression may hamper the correlation of PD-L1 expression with clinical benefit from immunotherapy. Moreover, PD-L1 expression may change during tumor progression; thus, the results may differ depending on the time of biopsy. Additionally, PD-L1 expression may be different in tissues from primary and metastatic tumors. Finally, no standardized cutoff value for PD-L1 expression has been validated, and some studies with anti-PD-1 agents have shown responses (even complete responses) in tumors not expressing PD-L1 62.

In gastric cancer, limited data relate tumor PD-L1 expression and the objective response rate to immunotherapy. In the phase 1b JAVELIN study, patients with tumors with higher expression levels of PD-L1 were more likely to exhibit tumor response during treatment with avelumab 23 in both the second-line and maintenance settings than those with lower tumor PD-L1 expression. Additionally, in the KEYNOTE-059 trial (cohorts 1 and 2) 19,63, patients with a PD-L1 expression of ≥ 1% had higher response rates to pembrolizumab (or pembrolizumab plus chemotherapy, for cohort 2). However, complete responses have been reported in patients with PD-L1-negative gastric cancers treated with pembrolizumab (cohort 1 of KEYNOTE-059). Interestingly, in the KEYNOTE-012 trial, among patients with PD-L1-positive tumors (PD-L1 expression of ≥ 1%), those with tumors with higher mononuclear inflammatory density scores were more likely to exhibit tumor response 17. However, despite this evidence, in the only randomized phase III clinical trial of anti-PD-1 immunotherapy in gastric cancer published so far (ATTRACTION-2), no differences in survival benefit were seen between patients with PD-L1-positive tumors and those with PD-L1-negative tumors 21. Additionally, in HCC, no association has been shown between tumor PD-L1 expression and the response rate to nivolumab 54. Therefore, PD-L1 expression does not currently seem accurate enough to guide treatment decisions regarding the use of ICP inhibitors in GI malignancies. Future studies should examine the molecular subtypes of gastric cancers that are more “immunogenic” and are thus more likely to respond to ICP inhibitors, such as the Epstein-Barr-virus associated type 64.

MSI-H has emerged as a potential predictor of immunotherapy response, regardless of the primary tumor site. Tumors with this molecular abnormality present a high mutational load and neoantigen formation, the latter of which may facilitate immune recognition and the activation of an antitumor response 58. Neoantigen formation has been found in many tumor types (Table 3), including several GI malignancies 65. Studies have shown that patients with MSI are more likely to respond to immunotherapy than those without MSI. Higher response rates to pembrolizumab have been found in patients with MSI-H gastric cancer in the KEYNOTE-012 (2 of 4) and KEYNOTE-059 (4 of 7) trials 17,19. Additionally, in a study assessing the activity of pembrolizumab in many MSI-H tumor types (including ampullary carcinoma, cholangiocarcinoma, pancreatic cancer, small bowel cancer and gastric cancer), 8 of 17 patients showed a tumor response to treatment, with durable responses seen in most of these patients 66. An additional study showed a clear correlation between MSI-H and a benefit from ICP inhibitors 22. Thus, in May 2017, the FDA approved pembrolizumab for the treatment of patients with MSI-H tumors that have been previously treated. Nevertheless, patients with non-MSI-H tumors have also responded to immunotherapy 19. In KEYNOTE-059 (cohort 2), 9% of patients with non-MSI-H tumors showed a tumor response to pembrolizumab. However, although the MSI-H status selects for patients likely to benefit from ICP inhibitors, prospective studies have shown that patients with non-MSI-H tumors may also respond to this treatment strategy.

Another predictive biomarker of the checkpoint inhibitor response, which is associated with but independent of MSI-H status, is a high tumor mutational burden. The rationale behind this biomarker is that the higher the tumor mutation burden is, the higher the probability of neoantigen formation. Therefore, other genetic conditions associated with high mutational tumor burdens, such as POLE mutations (germline and somatic), which are found in rare cases of endometrial 67 or GI malignancies 68, will likely predict a great benefit from ICP inhibitors.

Despite the clear ability of MSI-H to predict ICP inhibitor response, no perfect biomarker for selecting patients for immunotherapy has been found. Further development in this field is necessary for the accurate selection of patients for treatment with checkpoint inhibitors 69. Additionally, combined biomarker strategies are promising; such strategies are superior to single biomarkers for predicting the response to immunotherapy 70.

## PERSPECTIVES AND CONCLUSIONS

Advances in basic immunology have led to the discovery of PD-1, PD-L1 and CTLA4 as targets to promote the immune response against cancer cells. However, this new era in oncology is just beginning. New T cell inhibitory proteins (e.g., LAG-3, TIM-3 and VISTA) and T cell stimulatory molecules (e.g., ICOS/ICOSL and CD27/CD70) that are important regulators of immune surveillance have been discovered, and directed therapies are being developed 71. Additionally, some elements of the innate immune response, such as pathways inhibiting macrophages and natural killer cells, as well as the immunosuppressive enzyme IDO (indoleamine-2,3-dioxygenase), have been shown to be upregulated in cancer; thus, additional studies are warranted to assess the feasibility and activity of agents inhibiting such elements of the immune response 71. Moreover, trials evaluating the role of monoclonal antibody combinations targeting different inhibitory pathways are underway. Additionally, as immunotherapy resistance mechanisms are increasingly recognized, more effective drugs can be developed 69. Finally, progress in molecular engineering has led to the development of chimeric antigen receptor T cells (CAR-T cells) 72, which have demonstrated impressive results in acute lymphoblastic leukemia 73 and promising activity in early studies in GI tumors 74-76. 

Of equal importance, as the number of targetable molecules increases, we should be able to improve our capability to properly select patients for specific immune-based therapies. Clearly, the current methodology of assessing PD-L1 expression is not accurate enough to predict who will benefit from ICP inhibitors. Similarly, if we use MSI status, which is a rare biological phenomenon, as the sole criterion to select patients for treatment with anti-PD-1/PD-L1 inhibitors, most of the patients who would potentially respond to this treatment would not have access to these drugs. Thus, further developments in this field are necessary in order to maximize treatment efficacy.

In conclusion, the emerging landscape of anticancer immunotherapy is encouraging, and novel strategies are expected to dramatically change the standard of care for noncolorectal GI malignancies. Not only are new drugs awaited, but further developments in patient selection also appear very important in the pursuit of better outcomes for patients with GI cancers.

## AUTHOR CONTRIBUTIONS

Jesus VH, Felismino TC, Barros e Silva MJ, Souza e Silva V and Riechelmann RP. All authors contributed equally to the literature review, manuscript conceptualization and writing.

## Figures and Tables

**Table 1:** Overview of immune checkpoint inhibitor trials in esophageal cancer.

Study	Number of Patients	Treatment	Setting	Response Rate (Complete Response Rate) - %	Disease Control Rate - %	Progression-free Survival - months	Overall Survival - months	Treatment-related Grade 3-4 Toxicity - %
KEY-NOTE028 (11)	83	Pembrolizumab 10 mg/kg every 2 weeks	Squamous cell carcinoma (78%) Previously treated (96%)	30 (0)	39	1.8	7.0	17
ONO-3845 (12)	65	Nivolumab 3 mg/kg every 2 weeks	Squamous cell carcinoma (100%) Previously treated (100%)	17 (2)	42	1.5	10.8	26

**Table 2:** Overview of immune checkpoint inhibitor trials in gastric cancer.

Study	Number of Patients	Treatment	Setting	Response Rate (Complete Response Rate) - %	Disease Control Rate - %	Progression-free Survival - Months	Overall Survival - Months	Treatment-related Grade 3-4 Toxicity - %
Ralph C, et al. (26)	18	Tremelimumab 15 mg/kg every 3 months	Metastatic Second-line	5.6 (0)	22.2	2.8	4.8	-
Bang Y-J, et al. (27)	114	Ipilimumab 10 mg/kg every 3 weeks for 4 doses, then every 12 weeks	Maintenance after first line	1.8 (0)	33.4	2.7	12.7	22.8
		Placebo		7.0 (0)	47.4	4.9	12.1	8.9
KEYNOTE-012 (17)	39	Pembrolizumab 10 mg/kg every 2 weeks	Metastatic Previously treated (85%)	22.0 (0)	36.1	1.9	11.4	13
KEYNOTE-059 (18) Cohort 1	259	Pembrolizumab 200 mg every 3 weeks	Metastatic 2+ lines of treatment	12 (3)	27	2.0	5.5	18
KEYNOTE-059 (18) Cohort 2	25	Pembrolizumab (200 mg every 3 weeks) + CDDP + fluoropyrimidine	Metastatic Treatment-naive	60 (4)	80	6.6	13.8	16
KEYNOTE-059 (18) Cohort 3	31	Pembrolizumab 200 mg every 3 weeks	Metastatic Treatment-naive	26 (7)	36	3.3	20.7	23
Segal NH, et al. (38)	28	Durvalumab 10 mg/kg every 2 weeks	Previously treated	7.0 (-)	25.0	-	-	-
JAVELIN (23)	62	Avelumab 10 mg/kg every 2 weeks	Metastatic Second-line	9.7 (0)	29	1.5	-	9.9
JAVELIN (23)	89	Avelumab 10 mg/kg every 2 weeks	Metastatic Maintenance	8.9 (2.2)	57.3	3.0	-	9.9
CheckMate-032 (20) Group 1	59	Nivolumab 3 mg/kg every 2 weeks	Metastatic Previously treated (100%)	12 (2)	32	1.4	6.2	17
CheckMate-032 (20) Group 2	49	Nivolumab 1 mg/kg + ipilimumab 3 mg/kg every 3 weeks	Metastatic Previously treated (98%)	22 (2)	41	1.4	6.9	47
CheckMate-032 (20) Group 3	52	Nivolumab 3 mg/kg + ipilimumab 1 mg/kg every 3 weeks	Metastatic Previously treated (100%)	8 (0)	37	1.6	4.8	27
ATTRACTION-2 (21)	493	Nivolumab 3 mg/kg every 2 weeks	Metastatic 2+ lines of treatment	11 (0)	40.3	1.6	5.2	10
ATTRACTION-2 (21)	493	Placebo		0 (0)	25.0	1.4	4.1	4
KEYNOTE-061 (25)	592	Pembrolizumab 200 mg every 3 weeks		16 (4)$	-	1.5	9.1	14
		Paclitaxel (weekly)		14 (3)	-	4.1	8.3	35
JAVELIN 300 (24)	371	Avelumab 10 mg/kg every 2 weeks	Metastatic	2.2 (0.5)	22.2	1.4	4.6	9.2
		Chemoterapy#	Third-line (86%)	4.3 (0.5)	44.1	2.7	5.0	31.6

#Physicians' choice of chemotherapy (either paclitaxel or irinotecan).

$Combined positive score (CPS) ≥1%

**Table 3:** Frequency of microsatellite instability (MSI) in noncolorectal gastrointestinal malignancies.

Tumor	MSI-H$ Retrospective Series (%)	MSI-H$ TCGA (%) (62)
Esophageal adenocarcinoma	6.5 (77)	1.6
Gastric carcinoma	8.2-37.0 (78)	19.0
Cholangiocarcinoma	0.0-42.0 (79)	1.3
Pancreatic adenocarcinoma	0.3-22.0 (80-84)	0.0
Small bowel adenocarcinoma	7.6-28.0 (85-87)	-
Ampullary carcinoma	6.0-10.0 (88-90)	-
Hepatocellular carcinoma	11.0-16.0% (91,92)	0.8

$MSI-H: high-frequency microsatellite instability.
